# Passive immunization by recombinant ferric enterobactin protein (FepA) from *Escherichia coli* O157

**Published:** 2013-06

**Authors:** Seyed Mehdi Larrie-Bagha, Iraj Rasooli, Seyed Latif Mousavi-Gargari, Zohreh Rasooli, Shahram Nazarian

**Affiliations:** 1 Department of Biology, Shahed University, Tehran-Qom Express Way, Opposite Imam Khomeini's shrine, Tehran-3319118651, Iran; 2 School of Medicine, Shahed University, Tehran-Iran

**Keywords:** Enterohemorrhagic *Escherichia coli*, FepA, Vaccine, Passive immunization

## Abstract

**Background and Objectives:**

Enterohemorrhagic *Escherichia coli* (EHEC) O157:H7 has been recognized as a major food borne pathogen responsible for frequent hemorrhagic colitis and hemolytic uremic syndrome in humans. Cattle are important reservoirs of *E. coli* O157:H7, in which the organism colonizes the intestinal tract and is shed in the feces.

**Objective:**

Vaccination of cattle has significant potential as a pre-harvest intervention strategy for *E. coli* O157:H7. The aim of this study was to evaluate active and passive immunization against *E. coli* O157:H7 using a recombinant protein.

**Materials and Methods:**

The recombinant FepA protein induced by IPTG was purified by nickel affinity chromatography. Antibody titre was determined by ELISA in FepA immunized rabbits sera. Sera collected from vaccinated animals were used for bacterial challenge in passive immunization studies.

**Results:**

The results demonstrate that passive immunization with serum raised against FepA protects rabbits from subsequent infection.

**Conclusion:**

Significant recognition by the antibody of ferric enterobactin binding protein may lead to its application in the restriction of Enterobacteriaceae propagation.

## INTRODUCTION

*Escherichia coli* O157:H7 causes sporadic outbreaks of intestinal disease in man. It has achieved notoriety as the cause of a number of large food- and water-borne outbreaks (1–3). Enterohemorrhagic *Escherichia coli* (EHEC) O157:H7 is an emerging pathogen that causes acute human gastroenteritis and hemorrhagic colitis (4, 5). EHEC also causes disease in newborn calves and asymptomatically colonizes the gut mucosa of adult bovines, constituting the main reservoir for food and environmental contamination (6, 7). Cattle as well as other ruminants serve as a reservoir for *E. coli* O157:H7. In particular, surveys of beef and dairy cattle have demonstrated carriage rates less than 0.5 to greater than 2.0% (8). As a result of *E. coli* O157:H7 carriage in cattle, beef and dairy products often become contaminated and serve as a source of infection in outbreaks of *E. coli* O157:H7. In addition, it is associated with Shiga toxins (Stx), which can cause systemic complications including hemolytic uremic syndrome (HUS) and thrombotic thrombocytopenic purpura (TTP) affecting the kidneys and the central nervous system that may lead to death (9). The ability of the organism to survive in feed, water, soil, and manure has important implications for its persistence in cattle herds and contamination of water supplies and crops. Effective measures to reduce or eliminate *E. coli* O157:H7 in cattle will reduce not only food borne illness but also the risk of transmission of the organism into the environment (10).

A variety of treatment and prevention strategies to protect against *E. coli* O157:H7 are currently in development; these include toxin receptor analogues, passive antibody therapy, and vaccines to protect humans against the systemic effects of the toxin. Since an *E. coli* O157:H7 vaccine has not been developed or licensed for immunization of humans except for two vaccines that are currently in use in cattle, the most promising prevention strategies for *E. coli* O157:H7 focus on minimizing exposure to this pathogen (11–14).

Iron is an important micronutrient for virtually all living organisms, except lactic acid bacteria where manganese and cobalt are used in place of iron. The low solubility of the ferric form is a serious obstacle to microorganisms that require iron. Possession of iron uptake systems is known to be important in bacterial pathogenesis. Targeting surface-exposed, specific antigenic proteins that can induce antibody production to block uptake of vital elements such as iron into the bacterial cell is a strategy to combat or control pathogens. The iron-regulated outer membrane proteins (IROMPs) are appealing candidates as vaccine antigens because they are surface-exposed, antigenic, and may induce production of antibodies to block uptake of iron into the bacterial cell (15). Our previous report on immunogenicity of the membrane protein, FepA from *E. coli* O157:H7, indicated that the antibody produced therein could efficiently recognize and bind ferric enterobactin binding protein in the immunized mice challenged with higher doses of bacteria (16). Passive immunization is an appropriate therapeutic option against many organisms. Passive immunization with specific antibodies may have the potential to reduce *E. coli* O157:H7 pathogenesis in hosts (17).

In order to practically exercise the application scope of the immunity brought about by FepA, this study was designed to evaluate active and passive immunity against *E. coli*, *Shigella flexneri*, *Klebsiella pneumoniae*, and *Salmonella typhi* that share *fepA* gene homology of above 52%.

## MATERIALS AND METHODS

### Bacterial strains, plasmids, and culture media

*E. coli* Ol57:H7 (ATCC 43889), *Shigella flexneri* (NTCC 12678), *Klebsiella pneumoniae* (ATCC 13883), and *Salmonella typhi* (PTCC 1609) were used. The bacteria were cultured in Luria–Bertani (LB) broth or LB agar at 37°C for 24 hours media were supplemented, when required, with kanamycin (SIGMA, 40µg/ml). Sorbitol-MacConkey agar containing 0.5 mg/ml cefixime and 2.5 mg/ml potassium tellurite was used to test shedding of *E. coli* O157:H7. The plasmid pET28a(+) harbouring *fepA* gene was constructed in our laboratory (16) was used for expression of the protein. Bacterial strains were grown in LB broth at 37°C and were stored at －70°C in 20% glycerol. Nickel–nitrilotriacetic acid (Ni–NTA) agarose was from Qiagen (Valencia, USA). All other analytical grade chemicals and reagents were from Merck (Darmstadt, Germany).

### Animals and animals husbandry

Animal isolation rooms had individual floor drains. Floors and walls were washed once daily with water via a high pressure sprayer to minimize repeated challenge from bacterial shed through each animal's own feces. 100 male BALB/c mice, 8 weeks old, procured from the Razi Institute, Tehran, Iran were housed in clean standard and well-aerated conditions in the animal care facility at Shahed University. Research was conducted in compliance with the Animal Welfare Act and regulations related to experiments involving animals. The principles stated in the Guide for the Care and Use of Laboratory Animals were followed. Six male New Zealand White rabbits, 8 weeks old, each weighing about 1.5kg, were procured from the Pasteur Institute, Tehran-Iran. Four of these animals, chosen at random, were transported to and individually housed in containment rooms. Two rabbits served as control. One week prior to use, the isolation rooms were disinfected by fumigation with paraformaldehyde. Rabbits were fed once daily with a nutritionally balanced ration without antibiotics and water was available ad libitum. The animals were acclimated to the rooms and diet prior to inoculation.

### Expression and purification of FepA protein

The protein was expressed and purified as per our previous method described earlier (16). FepA was expressed in LB broth containing 50 mg/ml kanamycin by induction with 1 mM isopropyl b-D-thiogalactoside (IPTG) at 37°C for 4 h. The cells were harvested by centrifugation and resuspended in 0.3 ml of lysis buffer (50mM NaH_2_PO_4_, pH 8.0, 300mM NaCl, 0.2 mg/ml lysozyme). The samples were analyzed by SDS-PAGE. The recombinant FepA was purified by Ni–NTA affinity chromatography under native condition. The FepA protein was eluted by a stepwise procedure, using buffers containing 20, 40, 90 and 150 mM imidazole. The fractions were collected and analyzed by sodium dodecyl sulfate polyacrylamide gel electrophoresis (SDS-PAGE). Protein concentration was determined by the method of Lowry et al. (18) using bovine serum albumin (BSA) as a standard.

### Growth under iron-limiting conditions

The bacterial ability to grow in the presence of the iron chelator 2,2′-dipyridyl (Sigma-Aldrich) was determined by using tryptone soy broth supplemented with 1% NaCl as a basal medium. Production of compounds with siderophore activity was tested using the Chrome Azurol S (CAS) assay (19) in solid and liquid media. The assays were performed by spotting 10 µl of each bacterial culture obtained from a 3-day tryptone soy agar supplemented with 1% NaCl onto CAS agar. The siderophore levels produced by the strains on plates were expressed as the ratio of the orange halo diameter to the growth diameter after 72 h of incubation until 7 days post-inoculation. For siderophore detection in liquid media, supernatants from culture grown in tryptone soy broth supplemented with 1% NaCl containing 2,2′-dipyridyl were mixed with CAS supernatant solution and the absorbance of the mixture was measured at optical density of 630 nm (OD_630_).

### Immunization of rabbits

The week immediately prior to inclusion into the study, each animal was tested to assure negative for *E. coli* O157:H7 in fecal cultures. This test was also performed during the tenure of vaccination until the animals were challenged. The rabbits were also determined to be negative at day 0 for serum IgG against *E. coli* O157:H7 whole cells by Western immunoblotting. Four rabbits in the test group received four vaccinations of FepA protein each at 2 week interval. The animals were injected with 100µg of recombinant protein with complete Freund's adjuvant on day 1. The protein-complete Freund's adjuvant mix was divided into two equal parts. 50µg of the antigen/adjuvant mixture was injected subcutaneously and the other 50µg via muscular route. The subsequent injections were carried out in the preceding manner using recombinant protein with incomplete Freund's adjuvant, as the booster doses. The control group received PBS instead of the recombinant protein. Blood samples were collected through marginal ear vein ten days after the second, third and fourth injections. Sera from the blood samples were stored at －70 °C.

### Analysis of antibody response

Purified recombinant FepA protein was first diluted with coating solution to an optimal concentration (20µg/ml) in order to coat a 96-well plate. The resulting solution was then added into each well (100 µl per well) and incubated for 12–18 h at 4°C. To block the unoccupied sites, wells were washed three times with PBS plus 0.05% Tween 20 (PBS-T), and then incubated with 100 µl of PBS-T plus 5% skimmed milk for 1 h at 37°C. After washing the plates three times with PBS-T (100 µl per well), serial dilutions of each serum ranging from 1:2000 to 1:1024000 were added to the wells in triplicate and incubated at 37°C for 1 h. Plates were washed three times as described above. Antibodies binding to the antigen were detected using HRP goat anti-rabbit at 1:2000 dilutions. The colour was developed with orthophenylenediamine (OPD) for 30 min and the reaction was stopped with 2 M H_2_SO_4_. The plates were then read with a micro plate reader at 492 nm. Results were considered positive if the absorbance was at least double that of the control sera and the antibody titers were scored as the highest positive dilution.

### Western blot analysis

Protein samples were subjected to electrophoresis on a 9% SDS-PAGE gel and were then electroblotted onto a nitrocellulose membrane by Bio-Rad Mini Protean II System. The membrane was incubated in 3% BSA blocking buffer with gentle shaking for 1 h at room temperature. The membrane was then washed three times with PBS-T (PBS + 0.05% Tween-20, pH 7.4) and was then incubated with the diluted rabbit anti-FepA (1:100) serum for 1 h. After the PBS-T wash, the membrane was incubated with rabbit IgG conjugated with horseradish peroxidase (HRP) for 1 h at room temperature. The membrane was then washed three times in PBS-T. The membrane was visualized with diaminobenzidine (DAB) substrate until brownish bands were observed. Chromogenic reaction was stopped by rinsing the membrane twice with distilled water.

### Determination of lethal dose (LD_50_) in mice

The 50% lethal dose (LD_50_) was determined in the following manner: Bacteria at doses ranging from 10^4^ to 10^10^ CFU/0.5 ml mixed with equal volume of control rabbit sera were administered intraperitoneally to four groups of eight BALB/c mice per group. LD_50_ was estimated by the Probit method from the number of survivals on day 5.

### Passive immunization

Four bacterial strains viz, *E. coli* Ol57:H7, *Shigella flexneri*, *Klebsiella pneumoniae*, and *Salmonella typhi* at loads above their LD_50_ levels were mixed with equal volume of immunized rabbit sera and were then administered intraperitoneally to four groups of eight BALB/c mice. The development of disease in mice was monitored until they exhibited the signs of severe disease leading to death, such as hunched posture, poor mobility, and pilo erection.

### Challenging the immunized rabbits

In order to determine that the FepA-specific antibodies in immunized mice serum could reduce or prevent the *E. coli* O157:H7 shedding, subcutaneously immunized and non-immunized control mice were orally administered with 5×10^10^ CFU of *E. coli* O157:H7. Immunized mice sera mixed with live *E. coli* O157:H7 were orally administered to the rabbits. Prior to challenges each animal was tested to see negative for *E. coli* O157:H7 in fecal sample cultures. The animals were individually housed and permitted to have access to food and water ad libitum. Samples were collected daily from the centres of fresh faecal pats for 14 post-challenge days and were immediately processed. *E. coli* O157:H7 fecal shedding was monitored by adding 0.1g of feces to 1 ml of LB broth followed by incubation at room temperature for 1 h to allow the fecal pellets to soften. Serial dilutions of the supernatant were plated onto sorbitol-MacConkey agar plates containing 0.5 mg/ml cefixime and 2.5 mg/ml potassium tellurite. Plates were incubated overnight at 37°C and *E. coli* O157:H7 colonies were enumerated.

### Bactericidal assay of anti-FepA antibody

The bactericidal activity of anti-FepA serum raised in rabbits against each bacterial strain was assayed by the method given by Chen et al (20). The complement source for the experiment was fresh human serum from healthy individuals. Serial dilutions of the heat-inactivated anti-FepA serum were prepared. The bactericidal assay was performed by mixing 100 µl of bacterial suspension (approximately 2 × 10^6^ CFU) in PBS (containing 1 mM CaCl_2_ and 0.2 mM MgCl_2_) with 50 µl of heat-inactivated serum and incubating for 30 min at 4°C. About 30 µl of the complement containing serum was then added (1:10 diluted) to give a final concentration of 20% and the mixture was incubated overnight at 37°C in 96-well plates. The samples from wells were plated on nutrient agar plates and were incubated at 37°C overnight. The controls for this assay consisted of: (1) bacterial suspension + immune serum + heat-inactivated complement; (2) bacterial suspension + pre-immune serum + fresh complement; (3) bacterial suspension + pre-immune serum + heat-inactivated complement. Colony forming units (CFU) were calculated after incubation of nutrient agar plates and percent killing was calculated as follows: ((CFU from control – CFU from sample)/CFU from control) × 100.

### Statistical analysis

Data in each figure is a representative of three independent experiments expressed as the mean ± standard deviation (SD). All statistical analyses were performed using a SPSS 12.0 statistical program. ANOVA test was used to analyze the data from ‘‘Serum antibody variance’’ and student t-test for antibody responses. A value of P < 0.05 was considered as statistically significant.

## RESULTS

### Antigen and antibody preparation

**Fig. 1:**
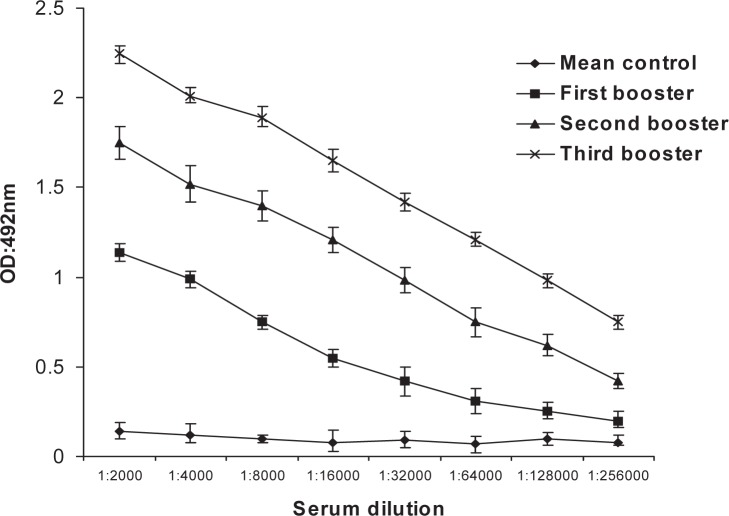
FepA-specific serum IgG following immunization.

We had already expressed FepA antigen in *E. coli* and purified the expression proteins by by His tag affinity chromatography for immunization purpose. The purified recombinant protein analysis was carried out with SDS-PAGE. A single band of approximately 85 kDa was revealed as reported earlier (16). Significantly, high antibody was detected in the test group compared to the control group injected with PBS (Fig. 1). The specificity of the recombinant protein was determined by Western blot analysis using FepA protein with antiserum. Production of recombinant FepA protein was reconfirmed in iron-restricted medium. The antibody was specifically reacted with the recombinant protein.

### Passive immunization with FepA antisera

This experiment was carried out in order to determine if serum from rabbits immunized with the FepA protein could be used to passively immunize BALB/c mice. The highest mortality of 50% was noted with *K. pneumoniae* at about 17×10^3^ LD_50_. The results are presented in Table 1.

**Table 1:** Passive immunization of rabbits with immunized serum.

Bacterial strains	Unimmunized animal serum + Bacteria	Immunized animal serum + Bacteria	×LD_50_	Survivals
*E. coli O157 H7*	8×10^9^	2.2×10^13^	2,750	75%
*Shigella flexeneri*	5×10^5^	4.3×10^11^	860,000	75%
*Klebsiella pneumoniae*	8×10^4^	1.3×10^9^	16,875	50%
*Salmonella typhi*	3×10^5^	2.6×10^11^	866,667	75%

### Challenging the immunized rabbits with *E. coli* O157:H7

**Fig. 2:**
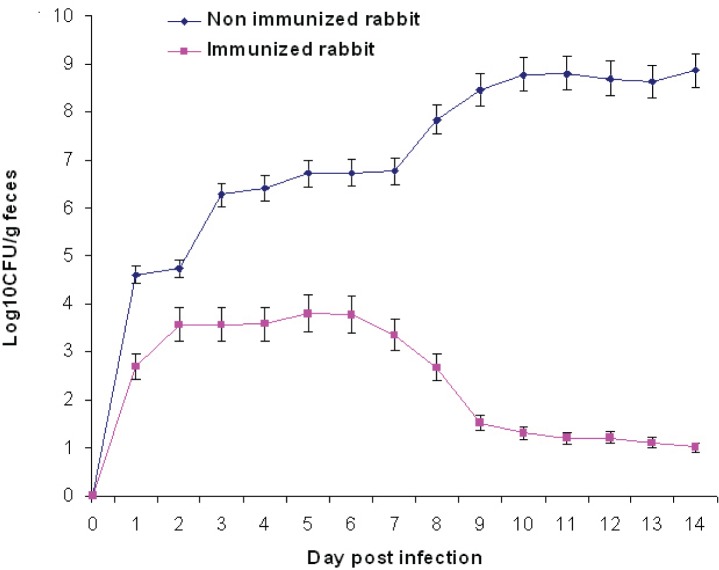
*E. coli* O157:H7 shedding in feces following subcutaneous administration of FepA protein in rabbit.

The unimmunized control rabbit shed high levels of *E. coli* O157:H7 (5.6×10^10^ CFU) over the two-week sampling period whereas shedding of immunized rabbit reduced gradually after seven days and was almost nil by the end of the second week (Fig. 2).

### Bactericidal assay of anti-FepA antibody

The bactericidal assay performed by mixing bacterial suspension with heat-inactivated anti-FepA serum resulted in above 95% bactericidal property with all four tested bacterial strains (Table 2).

**Table 2:** Bactericidal assay of anti-FepA serum.

Bacteria	Bactericidal activity %
*E. coli* O157 H7	94.72±7.17
*Shegella flexeneri*	100±0
*Klebsiella pneumonia*	98.98±1.14
*Salmonella typhi*	98.54±0

1/1000 dilutions of anti FepA serum were incubated with bacterial suspension in the presence of complement.

## DISCUSSION

Treatment of EHEC infection remains problematic since conventional use of antibiotics can enhance pathogenesis. EHEC can form characteristic attaching and effacing (A/E) lesions (21). Many bacteria deal with iron limitation by the secretion of siderophores, which are small organic compounds that bind iron very efficiently. The Fe^3 +^-siderophore complex is bound to specific receptors on the bacterial cell surface and internalized. The transport across the outer membrane requires energy, which is provided in *Escherichia coli* by the inner membrane protein TonB (22). Iron-regulated outer membrane proteins (IROMPs) are expressed under iron-deficient growth conditions. Several studies have shown that antibodies to IROMP have reduced the uptake of iron by blocking the binding of ferric complexes. The necessity for an effective intervention strategy to reduce faecal shedding of *E. coli* O157:H7 by ruminants as a means of reducing transference to humans is well recognized by researchers and is substantiated by apparently unremitting outbreaks worldwide (9, 23). *E. coli* O157:H7 excrete the catecholate siderophore enterobactin in response to iron deprivation, to solubilize iron prior to transport. Ferric enterobactin protein (FepA) is a Gram-negative bacterial OMP that transports ferric enterobactin. The bacterial iron limitation inducible OMPs hold a promise for vaccine development, since they expose a substrate binding domain at the cell surface, which must be conserved for function (24). If the immune system could be directed against this domain, an effective, broadly cross-reactive vaccine could possibly be developed. The structural data support the idea that the production of antibody against FepA may contribute to protection against Gram-negative bacteria with higher fepA gene homologies. In order to practically arrive at such a conclusion, further study of cross-immunity using recombinant FepA derived from each of the pathogens is suggested. In this study we demonstrated that a recombinant protein designated FepA could protect the animal from *E. coli* O157:H7 colonization. In order to achieve an optimal induction and powerful response, we applied booster doses at least three weeks after the first injection. The doses were gradually decreased to enhance affinity and to fully activate the B cell immune cascade. Finally fourth injection was intraperitoneal to activate clonal B cell effectively (25, 26). Accordingly a significant difference in the antibody titer was observed between sera of the immunize mouse and control groups. The periplasmic extraction of bacterial cells grown in iron restricted medium showed FepA production, while the cells grown in iron-rich medium did not produce FepA. These findings further support the idea that the mechanism of iron uptake may constitute an important virulence factor, which can help in establishing infection in the host. Here we show that passive immunization with serum from mice vaccinated with the FepA is able to protect animals from infection when administered prior to inoculation.

The bacterial counts determined in the bactericidal assay showed a significant decrease in the bacterial counts compared with controls. The 94.7–100% bactericidal property obtained (Table 2) emphasizes the role of humoral immunity against *E. coli* infection. Therefore growth inhibition of the bacterium was accomplished as a result of blocking the uptake of iron. This is in agreement with earlier observations where anti-IROMP antibodies significantly reduced *E. coli* septicemia and severity of gross lesions in turkeys (17). Our data showed that passive immunization strongly protected mice against EHEC challenge and immunized animals shed very low level of *E. coli* O157:H7 following infection (Fig. 2). These results demonstrate that antibodies alone are sufficient for providing protective immunity against *E. coli* O157:H7 infection, which may have implications for the development of future antibody based therapies. Active and passive immunization with FepA protein from *E. coli* O157:H7 can protect against infection with other bacteria such as *S. flexneri*, *K. pneumoniae*, and *S. typhi* further supporting the use of this antigen as a potential vaccine nominee. This level of reduction with recombination of antigen can be used as a novel strategy for developing a vaccine against *E. coli* O157:H7 shedding. This could also be a new strategy applicable to further researches in this field. Passive immunization that is a way to immunize host with pathogen-specific antibodies from diseases has been an attractive approach in controlling the spread of diseases in community.

In conclusion, the data demonstrate that active and passive immunization with FepA can prevent disseminated *E. coli* O157:H7 infection in rabbit model, and that a FepA vaccine may provide a viable immunization approach. This vaccine candidate is intended for use in cattle, to reduce the shedding of *E. coli* O157:H7 by cattle. It is speculated that the reduction of shedding in cattle will have a positive effect on the level of environmental contamination and so reduce contamination of produce.
